# GB Virus C Infection Is Associated with Altered Lymphocyte Subset Distribution and Reduced T Cell Activation and Proliferation in HIV-Infected Individuals

**DOI:** 10.1371/journal.pone.0050563

**Published:** 2012-11-29

**Authors:** Jack T. Stapleton, Kathryn Chaloner, Jeffrey A. Martenson, Jingyang Zhang, Donna Klinzman, Jinhua Xiang, Wendy Sauter, Seema N. Desai, Alan Landay

**Affiliations:** 1 Research and Medical Services, Iowa City VA Medical Center, Iowa City, Iowa, United States of America; 2 Departments of Internal Medicine, Microbiology and Immunology, The University of Iowa, Iowa City, Iowa, United States of America; 3 Department of Biostatistics, College of Public Health, The University of Iowa, Iowa City, Iowa, United States of America; 4 Department of Statistics & Actuarial Science, College of Public Health, The University of Iowa, Iowa City, Iowa, United States of America; 5 Department of Microbiology, Rush University, Chicago, Illinois, United States of America; University of Pittsburgh Center for Vaccine Research, United States of America

## Abstract

GBV-C infection is associated with prolonged survival and with reduced T cell activation in HIV-infected subjects not receiving combination antiretroviral therapy (cART). The relationship between GBV-C and T cell activation in HIV-infected subjects was examined. HIV-infected subjects on cART with non-detectable HIV viral load (VL) or cART naïve subjects were studied. GBV-C VL and HIV VL were determined. Cell surface markers of activation (CD38^+^/HLA-DR^+^), proliferation (Ki-67+), and HIV entry co-receptor expression (CCR5+ and CXCR4+) on total CD4+ and CD8+ T cells, and on naïve, central memory (CM), effector memory (EM), and effector CD4+ and CD8+ subpopulations were measured by flow cytometry. In subjects with suppressed HIV VL, GBV-C was consistently associated with reduced activation in naïve, CM, EM, and effector CD4+ cells. GBV-C was associated with reduced CD4+ and CD8+ T cell surface expression of activation and proliferation markers, independent of HIV VL classification. GBV-C was also associated with higher proportions of naïve CD4+ and CD8+ T cells, and with lower proportions of EM CD4+ and CD8+ T cells. In conclusion, GBV-C infection was associated with reduced activation of CD4+ and CD8+ T cells in both HIV viremic and HIV RNA suppressed patients. Those with GBV-C infection demonstrated an increased proportion of naive T cells and a reduction in T cell activation and proliferation independent of HIV VL classification, including those with suppressed HIV VL on cART. Since HIV pathogenesis is thought to be accelerated by T cell activation, these results may contribute to prolonged survival among HIV infected individuals co-infected with GBV-C. Furthermore, since cART therapy does not reduce T cell activation to levels seen in HIV-uninfected people, GBV-C infection may be beneficial for HIV-related diseases in those effectively treated with anti-HIV therapy.

## Introduction

Chronic T cell activation accompanies HIV infection and contributes to HIV-related pathogenesis, and CD4+ T cell activation is required for efficient HIV replication [1]–[4]. The extent of activation, measured by CD38 and HLA-DR co-expression on CD4+ and CD8+ T cells, correlates with HIV disease progression [3]; [5]; [6]. Persistent activation leads to activation induced cell death, which contributes to the depletion of CD4+ T cells during chronic HIV infection [2]; [3]; [7]; [8]. Although combination antiretroviral therapy (cART) lowers HIV viral load (VL) below the limit of detection in most recipients, and reduces activation markers on CD4+ and CD8+ T cells, the level of activation does not return to levels found in healthy, uninfected subjects [9]; [10]. The increase in T cell activation appears to contribute to an increased risk for cardiovascular, malignant and hepatic disease among treated HIV-infected people [11]; [12].

GB Virus C (GBV-C) is a human flavivirus tentatively assigned to the *Pegivirus* genus of the *Flaviviridae* [13]. GBV-C is capable of persistent infection and is lymphotropic, replicating in CD4+ and CD8+ T cells and in B cells (reviewed in [13]). Due to shared modes of transmission, GBV-C viremia is prevalent in HIV-infected individuals, occurring in up to 42% of some cohorts [14]. GBV-C infection is reported to be associated with prolonged survival in several, though not all cross sectional studies [15]–[21], and a meta-analysis of studies including 1,294 HIV-infected subjects found significantly prolonged survival in subjects with persistent GBV-C viremia (RH 0.41; 95% C.I. 0.23, 0.69) [22]. Among HIV-infected individuals receiving blood transfusions, an association was recently found between incident GBV-C infection and prolonged survival was [23]. Furthermore, among mothers co-infected with HIV and GBV-C, mother-to-child transmission (MTCT) of GBV-C was associated with an 87% reduction in HIV MTCT [24]. GBV-C does not replicate well *in vitro*; however, low levels of replication have been documented in lymphocyte and other cell culture systems, and co-infection of peripheral blood mononuclear cells (PBMCs) with GBV-C and HIV *in vitro* results in inhibition of HIV replication [16]; [25]–[27]. In contrast, GBV-C replicates very efficiently *in vivo*, with average GBV-C VL levels of more than one million genome equivalents/mL plasma in HIV-infected hosts [17]; [27]; [28].

Although GBV-C interferes with HIV replication *in vitro* [16]; [25]; [26]; [29] and GBV-C VL is inversely related to HIV VL *in vivo* [17]; [30], the extent of the effect on HIV VL does not appear sufficient to explain the survival benefit observed in many studies (reviewed in [31]; [32]). Recent studies suggest that GBV-C has numerous effects on T cell homeostasis that may influence HIV disease progression (reviewed in [32]). We examined the effect of GBV-C on T cell activation and proliferation in chronically HIV-infected subjects who were either HIV treatment naïve, or who were receiving cART and had nondetectable HIV VL for six or more months. In addition, specific T cell sub-populations (naïve, central memory (CM), effector (Eff), and effector memory (EM)) were assessed. GBV-C was associated with reduced T cell activation in subjects with HIV VL suppressed on cART and those not receiving cART. Furthermore, GBV-C was associated with a reduction in T cell proliferation and a corresponding increase in the proportion of naïve CD4+ and CD8+ T cells.

## Materials and Methods

### Study Design and Patients

HIV-1-infected subjects were prospectively recruited from the University of Iowa HIV/AIDS Clinic and enrolled in these studies following provision of written informed consent. The study was approved by the University of Iowa Institutional Review Board. HIV was diagnosed in all subjects by documentation of both antibody and HIV VL measurement. Heparin anticoagulated blood was shipped overnight to Rush University (Chicago, IL) for expanded flow cytometric characterization of T cell subpopulations, activation, proliferation, and HIV co-receptor expression. Demographic information including age, gender, race, ethnicity, HIV transmission mode, CD4 count, CD4 percent, HIV RNA concentration (Amplicor Monitor; Roche Diagnostics Systems, Branchburg, New Jersey, USA), and hepatitis B and C serologies were obtained from the medical record. The lower limit of detection for HIV VL was 48 copies/mL.

### GBV-C Viremia Testing

To determine GBV-C infection among HIV-seropositive patients, serum was prepared and stored at −70°C. RNA was extracted from serum and GBV-C RNA amplified and detected using a one-step real-time RT-PCR as described [27]. GBV-C was quantified using a synthetically transcribed GBV-C RNA with measured A260/280 to generate a standard curve as previously described [33]. All samples with a cycle threshold value of >35 were repeated using an independent RNA preparation.

### Immunophenotypic Analyses by Multiparameter Flow Cytometry

Upon arrival at Rush University, plasma was prepared by centrifugation of the anticoagulated blood for 15 minutes at 400×g, and aliquots were stored at −80 C until used for further analyses. The remaining cells were diluted in PBS w/o Ca^++^ and Mg^++^ and processed over Lymphocyte Separation Media (Mediatech, Manassas, VA). The interface containing PBMCs was collected, washed in PBS, resuspended in FACS buffer and stained for flow cytometric evaluation. Samples were acquired and a minimum of 35,000 cells were analyzed on a BD LSR2.0 flow cytometer using FACS Diva v6.1.3 software for each analysis. Fluorochrome conjugated antibodies against the following cell surface markers were used in these studies: CD3, CD4, CD8, CD38, CD45RA, HLA-DR, CCR7, Ki-67, CCR5, and CXCR4 (BD Biosciences). Activation was defined as the percentage of cells examined that expressed CD38 and HLA-DR for total CD4+ or CD8+ T cells, including individual T cell subsets: naïve (CD45RA (RA)+ and CCR7 (R7)+), central memory (RA−/R7+), effector (RA+/R7-) or effector memory (RA−/R7-). Proliferation was defined as the percentage of cells that expressed Ki-67. HIV co-receptor expression was defined as the percentage of cells expressing CCR5 or CXCR4 expression.

### Statistical Analysis

The percentage of CD38+/HLA-DR+, Ki67+, CCR5+, and CXCR4+ cells in total and naïve, CM, EM, and effector CD4+ and CD8+ T cells were used as outcomes in statistical analyses. Because there was a heterogeneity in the variance, a Kruskall-Wallace (KW) non-parametric test was used for each cell type to examine if there are differences between the 4 groups defined by HIV viremia (cART suppressed or cART naïve, HIV-S/HIV-V) and GBV-C viremia (G−/+). To examine for a consistent pattern in the 4 groups between the 4 cell types, O’Brien’s test of the mean rank was used to look for similar patterns in several types of cells [34]. Comparisons between G+/− groups were assessed for each subgroup defined by HIV viremia using two-sample t-tests without the homogeneity of variance assumption [35]. Data were analyzed for those not on therapy and HIV viremic (HIV-V) and those whose HIV viremia was suppressed with cART (HIV-S) stratified by GBV-C RNA status. A main effect of G (+/−) independent of HIV viral load (HIV-V [viremic] and HIV-S [suppressed]) was estimated using an additive linear model [36]. When the KW test was significant, nominal (unadjusted) p-values are reported. Differences in viral load and CD4+ cell counts were compared between the HIV-V and HIV-S groups using the Wilcoxon test. All the analyses were performed in R [37].

**Table 1:** Characteristics of study cohort.

	cART naive, GBV-C Positive n = 10	cART naïve, GBV-C Negative n = 21	Suppressed GBV-C Positive n = 14	Suppressed, GBV-C Negative n = 14
				
Age	36	40	47	46
Male	9	12	13	9
Race				
White	9	16	12	13
Black	1	5	2	1
CD4 cells/mm3	599	323*	548	520
log_10_HIV VL	4.10	4.86	NA	NA
log_10_GBV-C VL	7.88	NA	8.03	NA

cART = combination antiretroviral therapy. Suppressed = subject is on cART and has a nondetectable (suppressed) HIV viral load. *p<0.01; p-value for all other comparisons is >0.05.

## Results

### Study Participants

Sixty five subjects participated in the study. Four were excluded due to loss of virologic control on the date of sampling, one was excluded as he initiated cART in the month prior to testing, and one subject’s cells were delayed 24 hours during shipping, leaving 59 evaluable subjects. GBV-C viremia was detected in 41% (24) of the 59 remaining HIV-infected subjects. All twenty eight subjects on cART evaluated had nondetectable HIV VL values on cART for >6 months, and 31 subjects were cART naive. The 28 subjects on cART had been tested previously for GBV-C, with half (14) GBV-C viremic and the remainder non-viremic. Ten of 31 (32%) of the cART naïve subjects not previously tested had plasma GBV-C viremia detected. Based on clinical history, 2 cART naïve subjects were HIV infected for less than 6 months while the remaining 57 subjects were chronically infected.

The study cohort included 27% women (16/59), 85% (50/59) Caucasian, 15% (9/59) African American, and 7% (4/59) of Hispanic ethnicity. GBV-C viremic and non-viremic subjects were not significantly different in age, race, gender, or ethnicity (Table 1). Among those on cART with suppressed HIV VL, there was no difference in the duration of suppression between G+ and G- subjects (57 months vs. 63 months, p = 0.63). A nonsignificant difference in HIV VL was observed with the GBV-C viremic group (4.1 log_10_) lower than those without GBV-C viremia (4.1 log_10_ vs. 4.86 log_10_ respectively; p = 0.70, Wilcoxon rank-sum test). CD4 counts were not significantly different in subjects on cART (548 cell/mm3 in G+ vs. 520 cells/mm3 in G-); however, cART naïve subjects with GBV-C viremia had significantly more CD4 cells (599/mm^3^) than did those without GBV-C (323 cells/mm^3^; p<0.01) (Table 1). All subjects on cART had nondetectable plasma HIV RNA on the day blood was obtained (<48 copies/mL).

**Figure 1:**
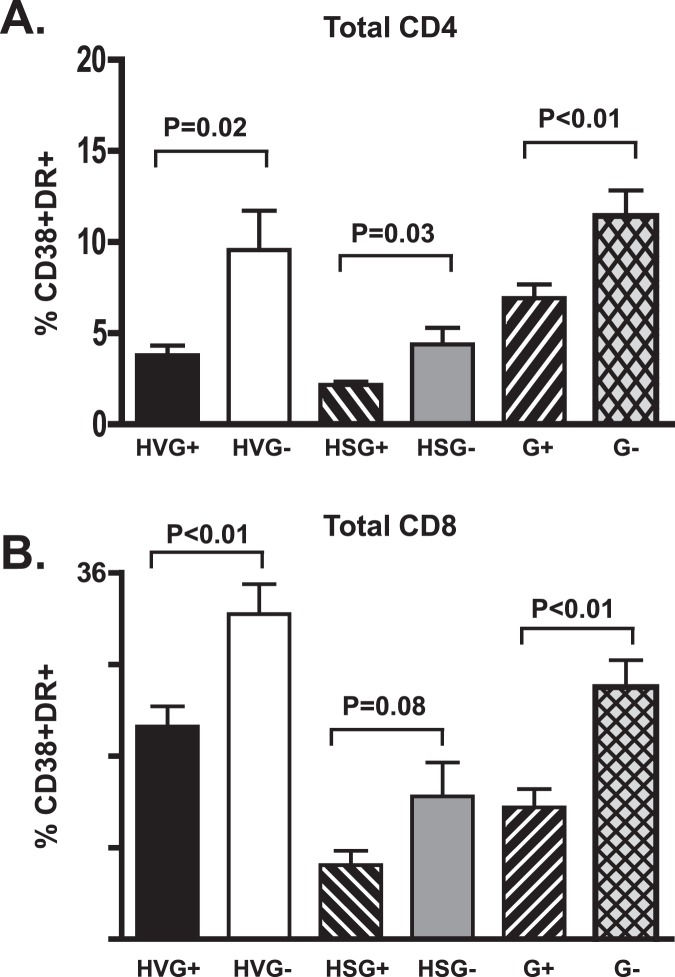
GBV-C is associated with reduced CD4+ and CD8+ T cell activation in HIV-infected individuals. The percentage of CD4+ (A) and CD8+ (B) T cells expressing CD38 and HLA-DR differed between GBV-C viremic (G+) subjects and those without GBV-C viremia (G-). Results of subjects not receiving combination antiretroviral therapy (HIV viremic; HV) or those with suppressed HIV RNA on therapy (HS) and statistical evaluation are provided.

### GBV-C is Associated with Reduced CD4+ and CD8+ T Cell Activation Markers

**Figure 2:**
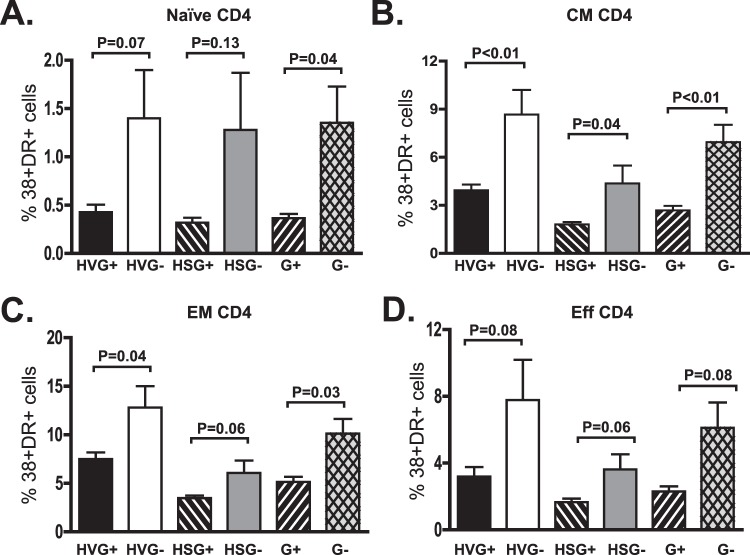
GBV-C is associated with reduced activation in CD4+ T cell subsets in treated and untreated HIV-infected individuals. The percentage of naive (A), central memory (CM; B), effector memory (EM; C) and effector (Eff; D) CD4+ T cells expressing CD38 and HLA-DR differed between GBV-C viremic (G+) subjects and those without GBV-C viremia (G-). Results of subjects not receiving combination antiretroviral therapy (HIV viremic positive; HV) or those with suppressed HIV RNA on therapy (HS) and statistical evaluation are provided.

**Figure 3:**
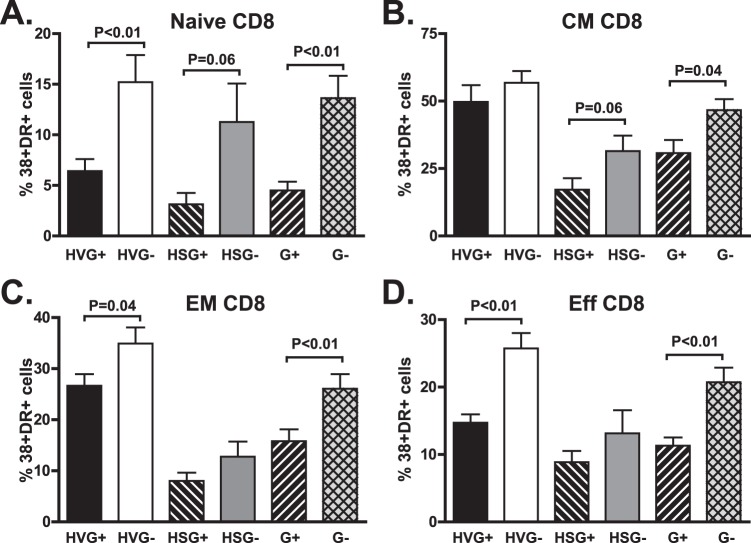
GBV-C is associated with reduced activation in CD8+ T cell subsets in treated and untreated HIV-infected individuals. The percentage of naive (A), central memory (CM; B), effector memory (EM; C) and effector (Eff; D) CD8+ T cells expressing CD38 and HLA-DR differed between GBV-C viremic (G+) subjects and those without GBV-C viremia (G-). Results of subjects not receiving combination antiretroviral therapy (HIV viremic; HV) or those with suppressed HIV RNA on therapy (HS) and statistical evaluation are provided.

Consistent with findings observed in recently HIV-infected subjects, GBV-C viremia was associated with a reduction in CD4+ and CD8+ T cell activation in subjects with HIV viremia as measured by CD38 and HLA-DR expression (Fig. 1, p = 0.02 and 0.002 respectively; Welsh test). In addition, G+ subjects with suppressed HIV VL on cART had significantly fewer CD38+/DR+ CD4+ T cells and a trend towards fewer activated CD8+ T cells (Fig. 1, p = 0.03, p = 0.08; Welsh test). Although GBV-C viremia was associated with lower activation in both CD4+ and CD8+ T cells, no significant correlation was observed between the GBV-C VL and the proportion of CD38+/DR+ cells, similar to a previous report (data not shown; [38]). When analyzing activation markers among the different T cell subpopulations, GBV-C viremia was associated most significantly with Naïve, CM, EM, CD4 T cells (Fig. 2), and with Naïve CM, EM and effector CD8 T cells (Fig. 3). Activation levels in CD4+ T cells, and to a lesser extent in CD8+ T cells were similar in G+ subjects not treated with cART and G- subjects fully HIV VL suppressed on cART (Figure 3).

### T Cell Subpopulations

**Figure 4:**
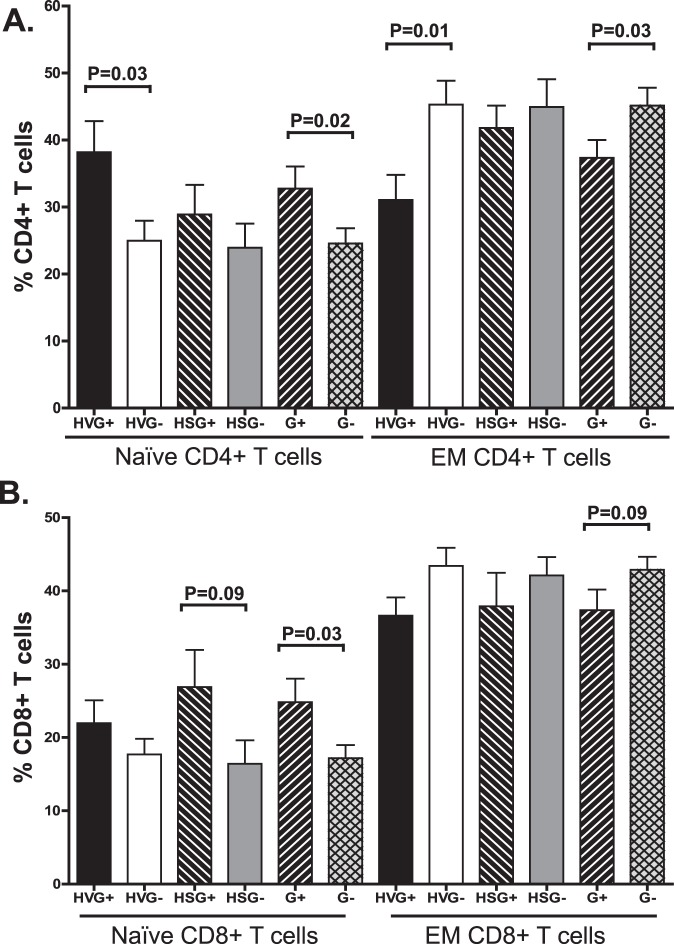
GBV-C is associated with altered distribution of naïve T cells in treated and untreated HIV-infected subjects. The proportion of naïve CD4+ (A) and CD8+ (B) T cells was higher in GBV-C viremic (G+) subjects compared to those without GBV-C viremia (G-). Results of subjects not receiving combination antiretroviral therapy (HIV viremic; HV) or those with suppressed HIV RNA on therapy (HS) and statistical evaluations are provided. In contrast, the proportion of effector memory cells (EM) was lower in G+ subjects.

The proportion of naïve CD4+ T cells was significantly higher among G+ subjects compared to G- (Fig. 4A). Although the difference was only significant for the HIV-V subgroup, a similar pattern was seen in HIV-S subjects. The increase in naïve CD4+ T cells was offset by a significant reduction in the EM CD4+ T cells (Fig. 4A). Similarly, among G+ subjects, the proportion of naïve CD8+ T cells was higher and there was a trend towards an increased proportion of and EM CD8+ T cells (Fig. 4B). Although the pattern for naïve CD8+ cells was consistent with naïve and cART treated subjects, the difference was greatest in subjects with suppressed HIV VL (Fig. 4B). There were no significant differences between G+ and G- subjects in the proportion of either CM or Eff CD4+ or CD8+ T cell populations (data not shown).

### GBV-C Viremia and T Cell Proliferation Markers

**Figure 5:**
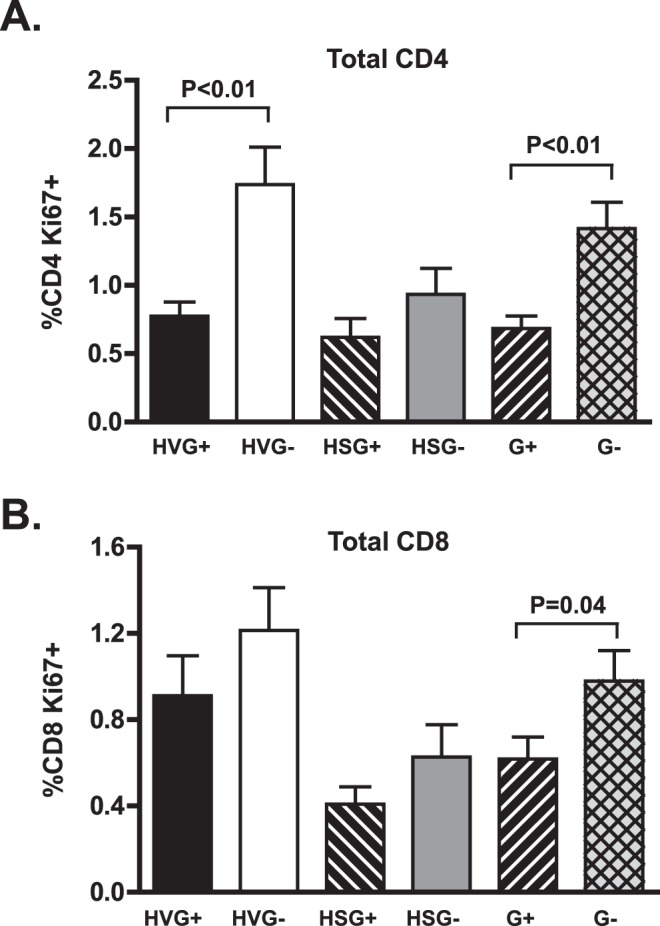
GBV-C is associated with reduced CD4+ and CD8+ T cell proliferation in HIV-infected individuals. The percentage of CD4+ (A) and CD8+ (B) T cells expressing the proliferation marker Ki67 differed between GBV-C viremic (G+) subjects and those without GBV-C viremia (G-). Results of subjects not receiving combination antiretroviral therapy (HIV viremic; HV) or those with suppressed HIV RNA on therapy (HS) and statistical evaluation are provided.

The association of GBV-C with reduced CD4+ T cell expansion following therapeutic IL-2 infusion [39], and the increased proportion of naïve T cells (Fig. 4) suggests that GBV-C is associated with altered proliferation. To determine if GBV-C is associated with a reduction in T cell proliferation (Ki-67) in HIV-infected subjects, we measured the percent of CD4+ and CD8+ cells (and each subpopulation) that expressed the proliferation marker Ki-67. Ki-67 positive CD4+ cells were significantly lower in G+ subjects compared to G- subjects after adjustment for HIV viremia status (Fig. 5, p<0.01). However, proliferation of CD8+ T cells were not different in G+ and G- subjects (p = 0.15). The expression of the Ki-67 proliferation marker frequencies were lower in G+ subjects for all T cell subsets combined and adjusted for HIV viremia status, but was only significant for the HIV-V group in CD4+ T cells. Individually the differences were only significant for the HIV-V CD4+ CM and Eff CD4+ T cell subpopulations (p = 0.01 and 0.05 respectively, data not shown).

### HIV Entry Co Receptor Expression

**Figure 6:**
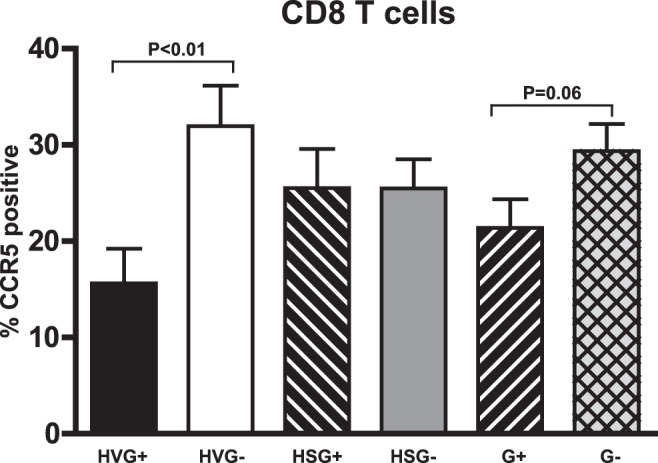
GBV-C is associated with reduced CCR5 expression on CD8+ T cells in HIV-infected subjects. The percentage of CCR5+ CD8+ T cells appeared to differ between subjects with HIV viremia (HV) with GBV-C viremia (HVG+) compared to those without GBV-C viremia (HVG-). This difference was not observed in subjects receiving cART.

GBV-C infection of PBMCs *in vitro* downregulates the HIV entry co-receptor CCR5 expression by reducing steady state mRNA concentrations [25]. GBV-C NS5A protein expression also reduces the surface expression and mRNA transcription of the HIV entry co-receptor CXCR4 in PBMCs and a CD4+ T cell line [40]. Previous clinical studies identified an association between GBV-C infection and a reduction in CCR5 and/or CXCR4 surface expression on CD4+ and CD8+ T cells, although results have varied among studies [41]–[43]. In this cohort, both the proportion of CD4+ T cells with CCR5 surface expression and the MFI of CCR5 on CD4+ T cells was lower in G+ subjects compared to G- in both the HIV-V and HIV-S subjects, although the decrease was too small to be significant in either group alone (data not shown). The frequency of CCR5 positive CD8+ T cells (p<0.01, Fig. 6) and the CCR5 MFI (data not shown) was significantly lower in G+ and HIV-V subjects. In contrast, there was no difference in CCR5 expression in the CD8+ T cells HIV-S group. High levels of CXCR4+ cells were present in all T cell subsets examined, and CXCR4+ CD4+ and CD8+ T cells were significantly increased in G+ subjects (data not shown). However, the clinical relevance of this finding is questionable, as the CXCR4 mean fluorescent intensity was not significantly different for any of the CD4+ or CD8+ T cell subsets and a high proportion (∼90%) of cells in both groups expressed CXCR4 (data not shown).

## Discussion

Persistent immune activation is a critical component of HIV pathogenesis (reviewed in [3]). Although T cell activation is reduced in successfully treated HIV-infected individuals, it remains increased relative to HIV uninfected control groups [9]; [10], and this is thought to contribute to increased morbidity and premature aging among those successfully treated with cART [10]; [12]; [44]. Understanding factors that modulate T cell activation may identify novel approaches to alter HIV disease progression.

Several lines of evidence suggest that GBV-C influences T cell homeostasis. First, GBV-C viremia is associated with a polarization towards a Th1 cytokine profile in a Sicilian HIV-infected clinical cohort [18], and this pattern of cytokine modulation is recapitulated in a cell culture model [33]. Secondly, GBV-C reduces activation-induced cell death and reactivation of latent HIV in peripheral blood lymphocytes obtained from HIV-infected people with suppressed HIV VL [27]. Third, GBV-C infection is associated with reduced CD4 T cell expansion in HIV-infected subjects treated with recombinant interleukin 2, suggesting an interaction between GBV-C and T cell proliferation [39]. Furthermore, recent studies demonstrated that the GBV-C envelope glycoprotein E2 inhibited IL-2 signaling and TCR-mediated CD4+ and CD8+ T cell activation *in vitro* [45]. Finally, GBV-C is associated with reduced T cell activation in a cohort of recently HIV-infected Brazilian subjects and cART treated U.S. subjects [42] [38]. However, this finding was not confirmed in a cohort of chronically HIV-infected subjects [46]. This may relate to methodological differences between the two studies. Specifically, the Brazilian study froze PBMCs after acquisition, and following thawing, the cells were stimulated with phorbol myristate acetate overnight prior to measuring cell surface CD38 expression, or intracellular IL-2 and interferon gamma expression by flow cytometry [47]. Thus, the Brazilian study did not assess baseline activation, but rather the ability of PBMCs to respond to activation stimuli following freeze-thawing. Our study directly assessed T cell activation without in vitro manipulation in chronically HIV-infected individuals.

In this study, among chronically HIV-infected subjects with viremia, GBV-C was associated with reduced activation of both CD4+ and CD8+ T cells (Fig. 1; [42]). Furthermore, GBV-C infection was associated with a significant reduction in CD4+ T cell activation in subjects with suppressed HIV VL on cART (Fig. 2; p = 0.05 for 4 types combined, and p = 0.03 for total CD4+). Thus, GBV-C viremia is associated with a smaller, but still significant, reduction in CD4+ cell activation in HIV-infected individuals successfully treated with cART. Finally, activation was reduced in all four T cell memory/naïve subsets (p<0.0001 in CM, Naïve, EM and Eff combined). A similar effect, though of lower magnitude, was seen CD8+ T cells (Fig. 3). GBV-C replicates in CD4+ and CD8+ T cells [48] and reviewed in [49], thus potentially explaining the association between G+ subjects and reduced activation in both cell types.

Although GBV-C was associated with significantly reduced activation, the level of the reduction was modest, especially in those with suppressed HIV VL. This is not surprising, as there are no pathological disease states clearly associated with GBV-C infection, and a more potent inhibition of T cell activation would presumably lead to immunosuppression. The relatively modest effect on T cells may reflect the fact that GBV-C is only present in a small percentage of T cells in peripheral blood (<5% of CD4+ cells) [48]. However, since activation markers are significantly reduced globally on T cell subsets, any effect that GBV-C may have on T cell activation would seem to require an effect of infected cells on uninfected bystander cells. It is unlikely that GBV-C and HIV infect the same CD4+ cell. However, co-infection of individual cells would not explain the global reduction in CD4+ T cell activation and proliferation that we observed. This global effect on T cell activation may reflect the induction of soluble factors (cytokines, chemokines), or the induction of double negative T cells that regulate T cell activation [25]–[27]; [33]; [38]; [45]. Alternatively, GBV-C viral proteins may provide inhibitory signals to bystander cells by virion-associated contact. Since GBV-C is produced by T and B lymphocytes and it is present in high concentrations in serum (average titer 5.45×10^7^ RNA copies/mL; [27], virtually all uninfected bystander cells will be in contact with GBV-C particles. Consistent with this hypothesis, CD4+ T cells expressing the GBV-C E2 protein inhibit HIV replication and reduce T cell activation in bystander cells when incubated with cell-to-cell contact *in vitro* [45]; [50]. Further studies to characterize the mechanism(s) by which GBV-C influences bystander T cell activation are underway.

These data are the first to demonstrate an association between GBV-C viremia and an increase in the proportion of naïve CD4+ and CD8+ T cells, and a reduction in proliferation. The reduction in proliferation is consistent with findings that CD4 cell expansion was impaired in G+ subjects following IL-2 infusion [39]. Furthermore, the reduced activation and proliferation observed in the G+ donors is consistent with an enrichment of the naïve T cell subpopulations (Fig. 4). Since cellular activation is required for efficient reverse transcription and viral spread [51]–[53], the reduced activation in G+ subjects may contribute to the reduced HIV VL observed in G+ subjects in some longitudinal studies [15]; [17]; [19], and in several, though not all cross-sectional studies [15]; [18]; [20]; [21].

Our data are consistent with earlier reports that GBV-C influences CCR5 expression *in vivo* [41]–[43] in that subjects with detectable HIV VL had reduced levels of CCR5 on CD8+ T cells (Fig. 6). In addition, and in contrast to an earlier report, CXCR4 expression was high in all subjects, but it was significantly increased in subjects with GBV-C viremia compared to subjects without viremia [43].

### Conclusions

In summary, GBV-C viremia was associated with reduced activation and proliferation, an increased proportion of naïve CD4+ and CD8+ T cells, and reduced CCR5 expression on CD8+ T cells. The relationship between GBV-C and these variables was consistent in subjects in which HIV VL was suppressed by cART, and in treatment naïve groups. Since cART does not reduce activation levels to that seen in HIV-uninfected populations, GBV-C may provide clinical benefit to subjects effectively treated with cART. Understanding the mechanism(s) by which GBV-C might diminish T cell activation may provide novel approaches to address the persistent T cell activation observed in HIV-infected people.
